# Gene Prioritization for Imaging Genetics Studies Using Gene Ontology and a Stratified False Discovery Rate Approach

**DOI:** 10.3389/fninf.2016.00014

**Published:** 2016-04-07

**Authors:** Sejal Patel, Min Tae M. Park, The Alzheimer's Disease Neuroimaging Initiative, M. Mallar Chakravarty, Jo Knight

**Affiliations:** ^1^Campbell Family Mental Health Research Institute, Centre for Addiction and Mental HealthToronto, ON, Canada; ^2^Institute of Medical Science, Faculty of Medicine, University of TorontoToronto, ON, Canada; ^3^Cerebral Imaging Centre, Douglas Mental Health University Institute, McGill UniversityVerdun, QC, Canada; ^4^Schulich School of Medicine and Dentistry, Western UniversityLondon, ON, Canada; ^5^Department of Psychiatry, McGill UniversityMontreal, QC, Canada; ^6^Department of Psychiatry, University of TorontoToronto, ON, Canada; ^7^Biostatistics Division, Dalla Lana School of Public Health, University of TorontoToronto, ON, Canada; ^8^Lancaster Medical School and Data Science Institute, Lancaster UniversityLancaster, UK

**Keywords:** magnetic resonance imaging (MRI), genome wide association study (GWAS), gene ontology network, Alzheimer's disease (AD), stratified false discovery rate (sFDR), imaging genetics

## Abstract

Imaging genetics is an emerging field in which the association between genes and neuroimaging-based quantitative phenotypes are used to explore the functional role of genes in neuroanatomy and neurophysiology in the context of healthy function and neuropsychiatric disorders. The main obstacle for researchers in the field is the high dimensionality of the data in both the imaging phenotypes and the genetic variants commonly typed. In this article, we develop a novel method that utilizes Gene Ontology, an online database, to select and prioritize certain genes, employing a stratified false discovery rate (sFDR) approach to investigate their associations with imaging phenotypes. sFDR has the potential to increase power in genome wide association studies (GWAS), and is quickly gaining traction as a method for multiple testing correction. Our novel approach addresses both the pressing need in genetic research to move beyond candidate gene studies, while not being overburdened with a loss of power due to multiple testing. As an example of our methodology, we perform a GWAS of hippocampal volume using both the Enhancing NeuroImaging Genetics through Meta-Analysis (ENIGMA2) and the Alzheimer's Disease Neuroimaging Initiative datasets. The analysis of ENIGMA2 data yielded a set of SNPs with sFDR values between 10 and 20%. Our approach demonstrates a potential method to prioritize genes based on biological systems impaired in a disease.

## Introduction

Imaging genetics is a burgeoning field that seeks to understand the association of neuroimaging-based phenotypes, such as structural, functional (Thompson et al., 2010), and diffusion imaging-based metrics, (Patel et al., 2010) with genetic variations. Candidate gene studies were initially the method of choice for understanding gene function in humans, and successfully identified genes involved in Mendelian diseases; however, such studies have had less success for complex genetic disorders, with many novel findings failing to replicate in further studies (Hirschhorn et al., 2002). Reasons for such failures include a lack of power to identify the small effect sizes typically involved in complex traits, as well as a lack of knowledge about which genes are appropriate to study (Tabor et al., 2002; Ioannidis, 2005). Around 2007, genome-wide association studies (GWAS) began to make inroads as an efficient method for identifying variants associated with complex disease.In this approach, approximately one million single nucleotide polymorphisms (SNPs) across the whole genome are interrogated simultaneously, hypothesis-free (Wellcome_Trust_Case_Control_Consortium, 2007). However, due to the large burden of multiple testing correction in a GWAS, a *p*-value of 5 × 10^−8^ or less, roughly equivalent to a *p* = 0.05 after Bonferroni correction for half a million independent variants, is generally required for a SNP to be recognized as significantly associated with a trait (Dudbridge and Gusnanto, 2008). Given the polygenic nature of complex traits and low effect sizes associated with these traits, large sample sizes are required to achieve adequate statistical power. Recently, a large imaging genetics study named ENIGMA (Enhancing NeuroImaging Genetics through Meta-Analysis) was undertaken, in which 21,000 subjects were included in a GWAS in order to identify genetic variants with association to hippocampal volume (Stein et al., 2012). While this study was a landmark demonstration for the use of imaging genetics techniques to investigate brain structures, it is not plausible for individual investigators to obtain such large sample sizes for their studies.

Various approaches have been described to reduce the multiple testing burden for large scale GWAS. One such approach is to control for the false discovery rate (FDR), rather than the family-wise error rate (FWER) (Benjamini and Hochberg, 1995). Where the family-wise error rate identifies the probability of one type 1 error from the total tested hypotheses, FDR calculates the proportion of expected type 1 errors. The use of FWER is more stringent compared to the use of FDR leading to less type 1 errors however the power associated with this method is lower, limiting the chance of detecting potential new discoveries. The differences are highlighted by an example from Benjamini and Yekutieli (2005) based on an QTL (quantitative trait locus) linkage analysis demonstrating a FDR threshold set at 0.05, corresponded to a FWER threshold of 0.64. However the utilization of FDR is more relevant in an exploration analysis on a dataset where there are likely to be multiple true positives (Sun et al., 2006). Furthermore FDR is useful as a screening method in GWAS with multiple phenotypes and covariates (Sun et al., 2006). Another suggested use of FDR is for types of studies where there is less concern on making type 1 errors. In our study our goal is to identifying loci which we would attempt to replicate rather than making strong claims of causality in the first instance, hence an exploratory approach.

Stratified false discovery rate (sFDR) is an extension of the FDR control approach, where the false discovery rate is controlled in distinct subsets (strata) of the data, one or more of which are believed to have a higher prior probability of being associated with the trait of interest. Strata are defined based on prior information such as linkage analysis, candidate gene studies, or biological pathways (Sun et al., 2006, 2012). An example of this approach by Sun et al. (2012) investigates the susceptibility to meconium ileus (severe intestinal obstruction) in individuals with cystic fibrosis by prioritizing a set of genes involved in the apical plasma membrane. In this article, Sun et al. (2012) selected strata defined by Gene Ontology (GO) terms. GO is a biomedical ontology database, which contains structured vocabulary terms known as GO terms designed to describe protein function (Ashburner et al., 2000). In more complex traits, this approach may not be refined enough for the proper stratification of data in sFDR. There are different approaches that can be used to prioritize genes depending on how well the phenotype is characterized. Sun et al. (2012) prioritized genes for stratification based on the single GO term “apical plasma membrane” to investigate targeted genetic modifiers of Cystic Fibrosis. However, applying that similar approach to complex genetic diseases such as AD may not be as effective. Utilizing a biological domain gene prioritization approach via GO will allow us to capture a wider group of potential genetics variants within biological systems associated with AD.

There have been other approaches designed to create lists of candidate genes based on previous findings. One example is the work by Linghu et al. (2009) which focuses on multiple diseases and the use of genomic features from a variety of databases. Similarly, Chen et al. (2007) uses GO with a combination of genomic databases to prioritize human disease candidate genes by utilizing information from mouse phenotype ontology and extracting mouse ortholog of human genes. Based on current utilization of GO in gene prioritization, our approach is novel in a number of aspects; (1) The gene seed list is based on GWAS meta-analysis results. (2) The use of GO allows us to take a biological system approach to investigate a disease of interest. By taking advantage of the biological process ontology we can feed in our gene list from previous GWAS results and identify common biological process domains which have not been seen in current gene prioritization methods using standard GO terms. Instead of investigating the disease as one system, our prioritization technique allows us to explore candidate genes within different biological systems implicated within the disease phenotype. (3) The use of sFDR allows us to stratify SNPs within each biological process group and implement it to GWAS data to identify if the set of SNPs within a biological process group is associated with the disease phenotype.

Another common use for GO terms is to use a list of significant genes from association analysis and identify enrichment of GO terms annotated to those genes. Commonly used tools for GWAS approach include WebGestalt, INRICH and Aligator. WebGestalt (WEB-based GEne SeT AnaLysis Toolkit) is a functional enrichment tool that utilizes different publically available resources, not only GO, but other genomic databases such as phenotype ontologies and protein-protein interaction datasets. Therefore a set of genes from an association analysis can be uploaded to WebGestalt, and the gene list is compared to genes in pre-defined functional categories. Similarly, INRICH (Interval-based Enrichment Analysis Tool for Genome Wide Association Studies) and Aligator (Association LIst Go AnnoTatOR) assess enrichment for GO terms based on a list of either candidate genes or SNPs (Holmans et al., 2009; Lee et al., 2012). Both INRICH and Aligator differ from WebGesalt by determining if linkage disequilibrium (LD) independent associated regions show an enrichment of specified characteristics, primarily pathways defined by GO terms. An example of an application is the paper by Lotan et al. (2014) which uses an array of neuroinformatics tools to analyze common and distinct genetic components associated in six neuropsychiatric disorders. GO was used to identify which biological domains were enriched within the gene list that was common among the neuropsychiatric disorders (Lotan et al., 2014). Studies similar to Lotan et al. (2014) has utilized GO for enrichment of genes once results of genetic variants have been obtained in association with a phenotype (Miyazaki et al., 2009; Jones et al., 2010; Anney et al., 2011). We have taken a reverse approach where we are using GO to build a biological system network based on genes which have already been associated with previous GWAS studies. The identification of the biological network allows us to explore other genes within a common biological process in association with the phenotype.

We demonstrate our method's efficacy by investigating the association between genetic variants and hippocampal volume in both the ENIGMA2 and Alzheimer's Disease Neuroimaging Initiative (ADNI1) dataset. The dominant symptom of Alzheimer's Disease (AD) is dementia, where memory, reasoning, and thinking are all impaired. The hippocampus plays a key role in cognitive functioning, influencing processes such as learning and the ability to make new memories (Braskie et al., 2013). Further, in considering the neurodegeneration of medial temporal lobe structures, the changes in hippocampal structure are considered to be one of the strongest quantitative phenotypes associated with AD and can often be used to predict cognitive decline in AD patients (Braskie et al., 2013). A particular biological system of interest that we used to prioritize our genes is the transport system which plays a key role in AD. For example the PICALM (Phosphatidylinositol-binding clathrin assembly protein) gene has been hypothesized to be involved in the transport of Aβ (amyloid beta plaque) in the blood stream (Baig et al., 2010) and Aβ clearance (Schjeide et al., 2011). Schjeide et al. (2011) demonstrated risk alleles for PICALM to be associated with reduced levels of Aβ in the cerebrospinal fluid of AD patients compared to controls.

This article presents a novel, systematic method to determine the optimal stratification of SNPs for sFDR analysis. We employed GO alongside previous GWAS findings, and applied our method to the ENIGMA2 and ADNI1 (Supplementary Section 2) data, both dataset containing AD individuals. Our method reduces the multiple testing correction burden with the potential to discover novel biomarkers in imaging genetics. Useful not only for new genetic studies, our tool is highly applicable to mining already existing GWAS data and improving the integration of publically available bioinformatics resources such as GO with imaging genetics studies.

## Materials and methods

**Figure 1:**
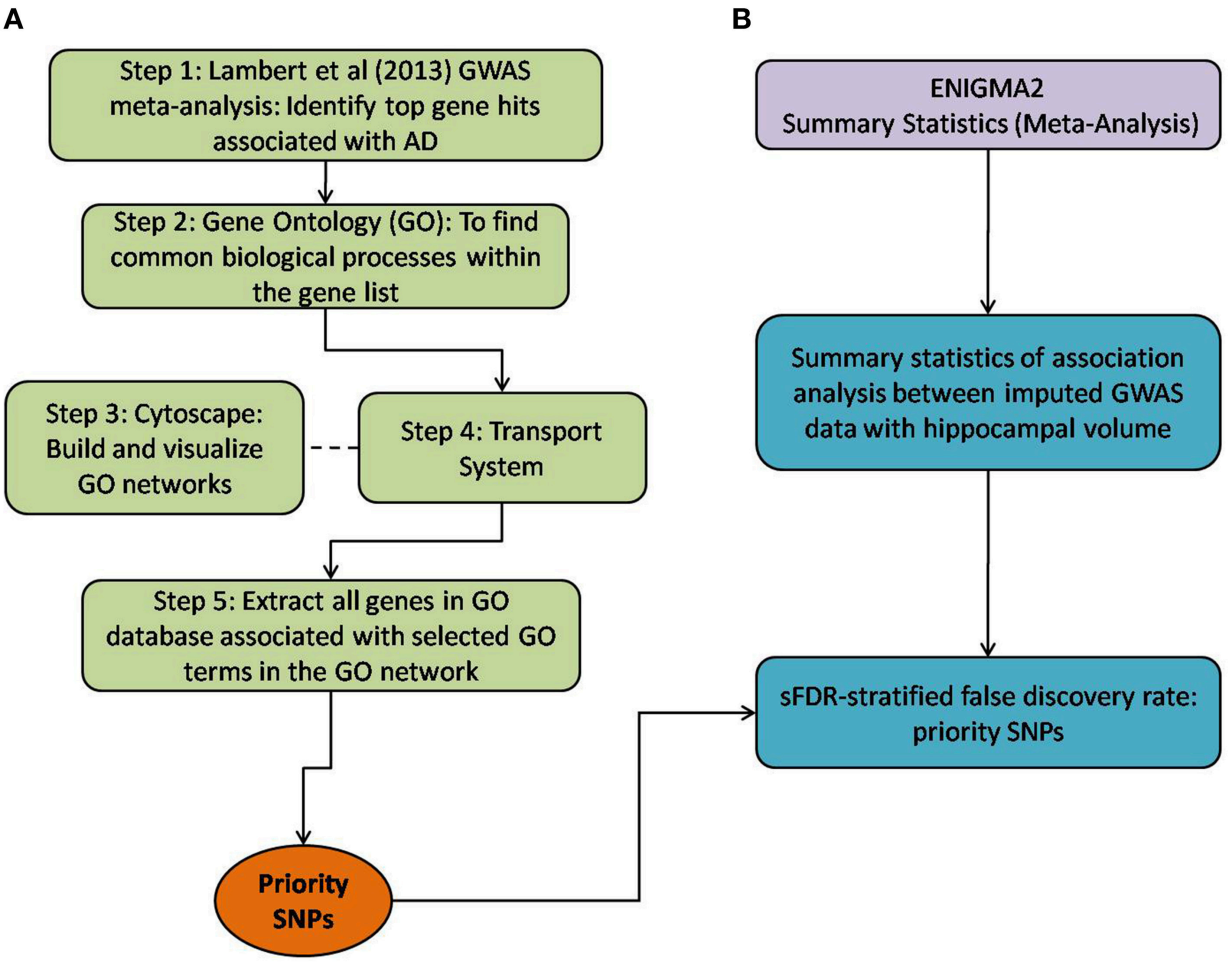
**Method overview of both the selection of priority SNPs and association testing analysis between ENIGMA2 GWAS summary statistics**. **(A)** Steps taken to select for priority SNPs. Gene hits from a meta-analysis by Lambert et al. (2013) were used as a starting point (Step 1) and GO was then used to identify common biological processes within the gene hits (Step 2). Cytoscape was used to build and visualize common biological process networks – in this case the “transport system” network was selected (Step 3 and Step 4). All genes from the selected GO terms in the network were extracted to form the priority list of SNPs. sFDR was then implemented with the priority SNPs. **(B)** Shows the utilization of ENIGMA2 summary statistics of GWAS meta-analysis on hippocampal volume.

Our methodological approach (Figure 1 highlights the overall steps) was applied to two different datasets, ENIGMA2 summary statistics and the ADNI1 dataset. In this paper, we focus on applying our method on the ENIGMA2 dataset. Further details of using our method with the ADNI1 dataset is documented in the Supplementary Section 2.1 and Figure S1.

### Alzheimer's disease (AD) model: selecting priority list of genes

Below, we detail how we assembled a list of priority genes, derived comprehensive gene networks based on these so-called “seed” genes, and pruned these networks appropriately. Our priority SNPs were selected from genes involved in biological systems associated with AD. Figure 1 outlines the entire process followed, including SNP selection (Figure 1A) and the utilization of ENIGMA2 summary statistics on GWAS meta-analysis of hippocampal volume (Figure 1B).

**Figure 2:**
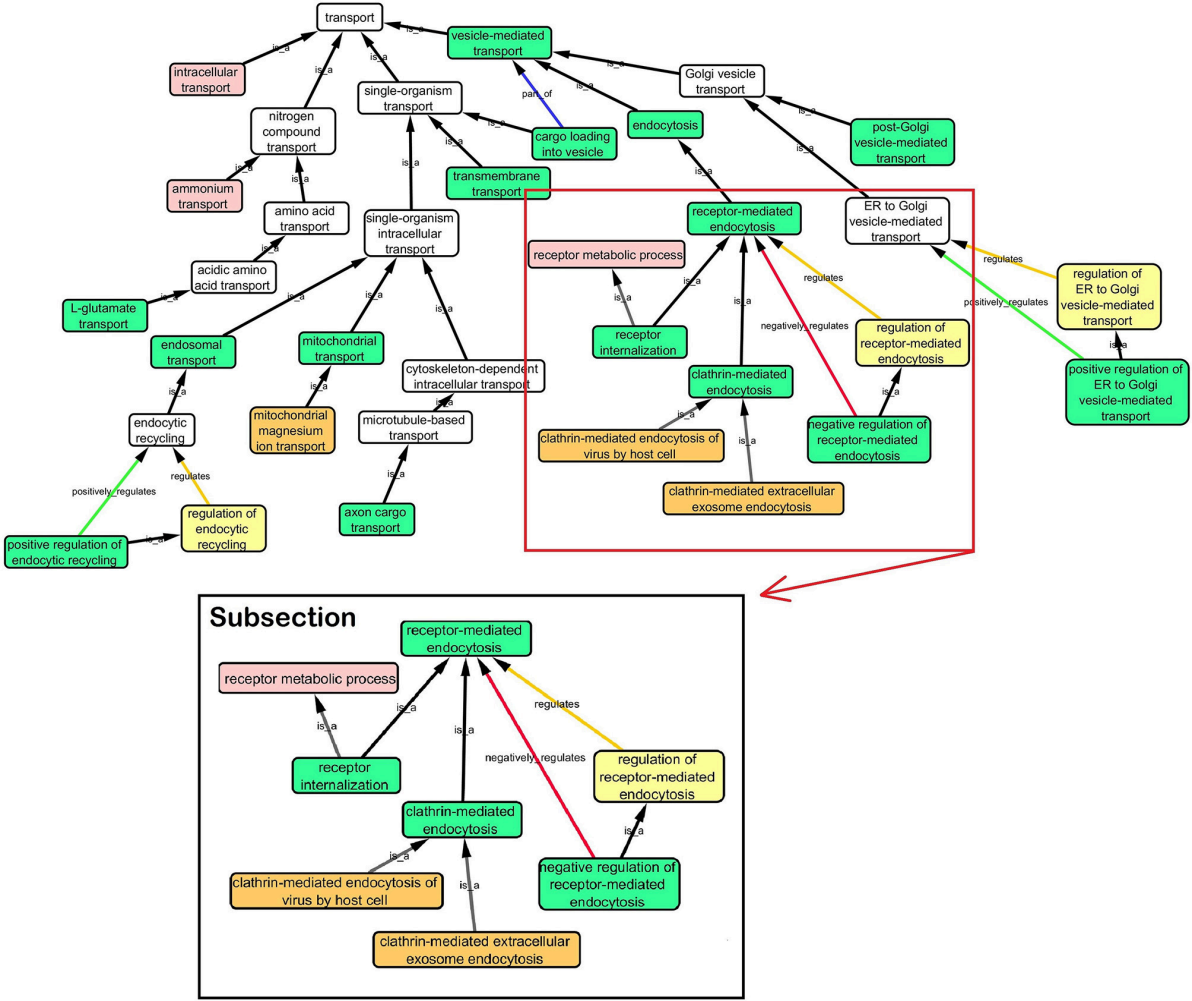
**Sample of initial transport system network with selected GO terms before pruning**. A subsection is selected to show how the criteria was used to prune the complex GO network. Pruning steps are shown in Figure 3.

**Figure 3:**
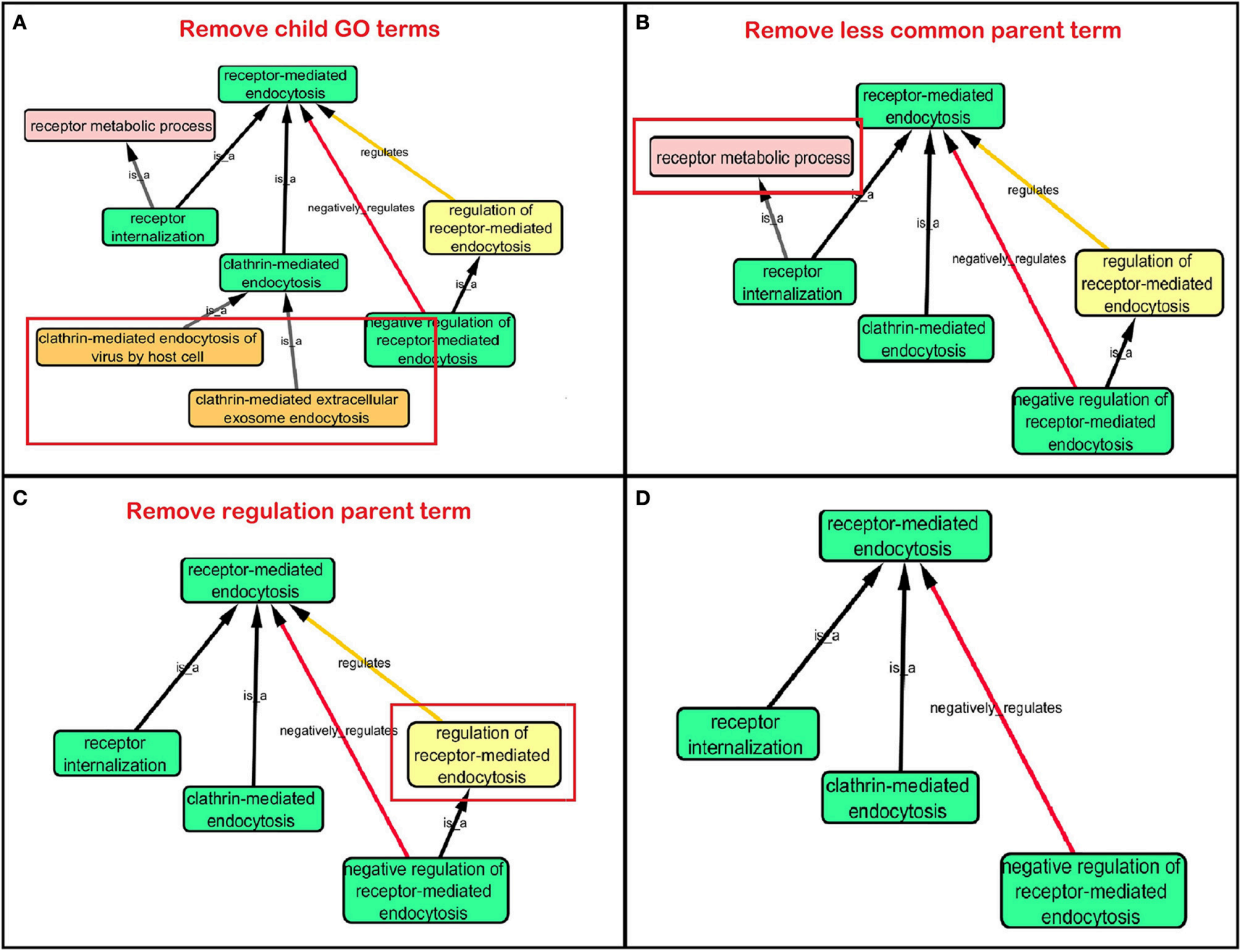
**Criteria used to prune a complex network**. Green box: Selected GO terms that are associated with a gene identified from Lambert et al. (2013). Orange box: Child terms of selected GO terms. Pink box: Less common parent term only associated with one selected child GO term. Yellow box: Regulation GO terms that do not specify positive or negative regulation. **(A)** Child terms of selected GO terms were removed. **(B)** A less common parent GO term (receptor metabolic process) which has one selected child GO term (“receptor internalization”) is removed because “receptor-mediated endocytosis” is a parent term for both selected GO terms “receptor internalization,” and “clathrin-mediated endocytosis.” **(C)** Regulation terms that do not specify the type of regulation is removed because selected GO term “negative regulation of receptor-mediated endocytosis” is more descriptive than the parent GO term “regulation of receptor-mediated endocytosis.” **(D)** A sample of a pruned network after following the criteria in Figures 3A–C.

**Figure 4:**
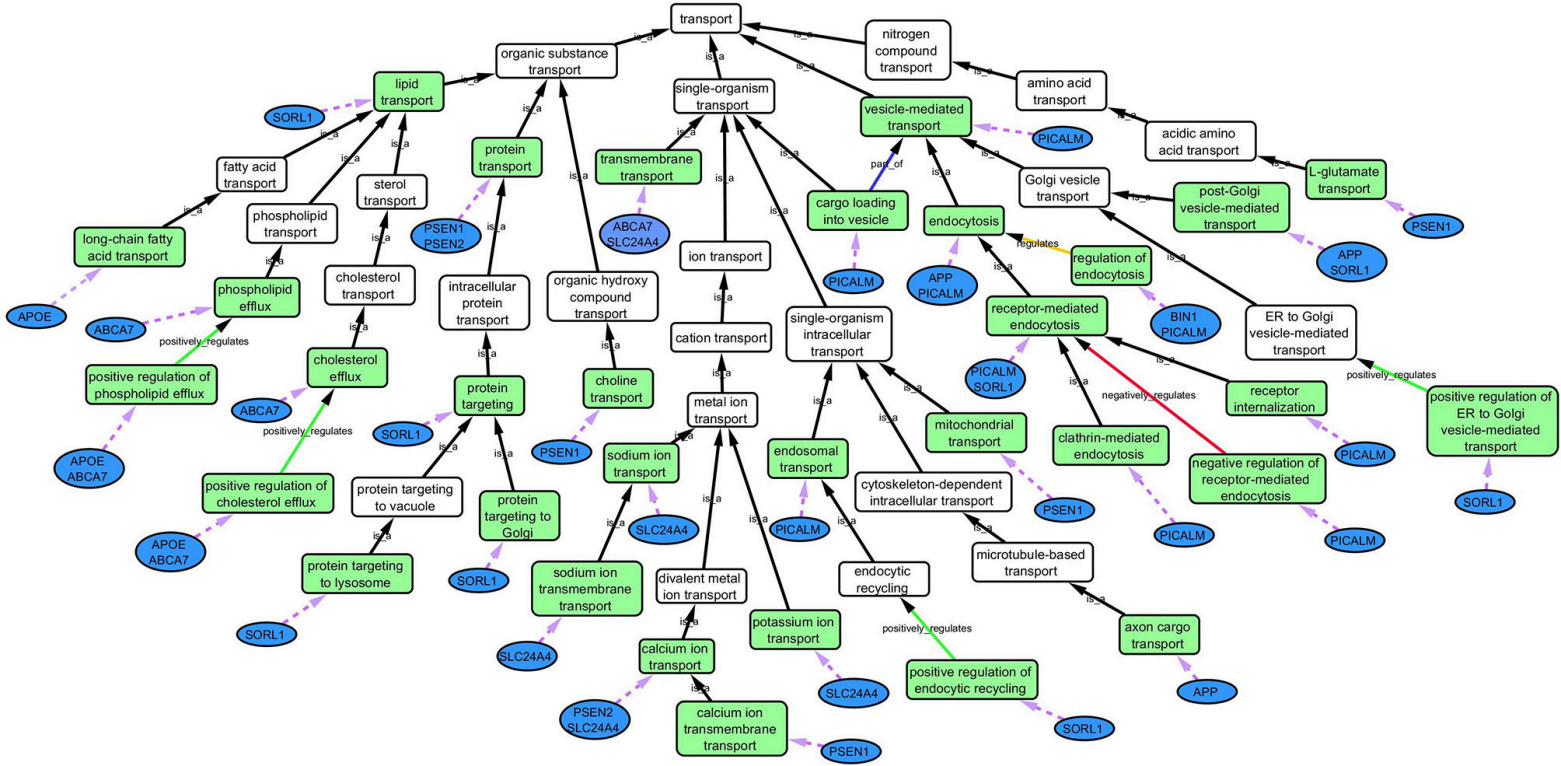
**Gene Ontology (GO) biological process network of the “transport system” in association with AD**. Green boxes are GO terms that are associated with the specific genes (blue ovals) connected by purple dotted line. White boxes are intermediate parent GO terms related to the selected GO terms (green boxes). Black arrows represent “is_a” relationship between the GO terms and its parent term; blue arrows shows a “part _of” relationship; orange arrows, a “regulation” relationship; green arrows, a “positive_regulation” relationship and red arrows, a “negative_regulation” relationship.

**Step 1:** Twenty-one hits from a previous meta-analysis of AD GWAS signals were used as a starting point to identify top gene hits (Lambert et al., 2013). In addition we added amyloid precursor protein (*APP*) (Goate et al., 1991), Presenilin-1 (*PSEN1*) and Presenilin-2 (*PSEN2*) (Cruts et al., 1998) to our gene list based on association with familial form of AD. Furthermore rare variants within these gene regions also increase the risk of late onset AD (Cruchaga et al., 2012).
**Step 2:** Gene Ontology (GO) (refer to Box 1, data release July 1, 2015) was used to group genes, and subsequently to derive common biological process networks using a three step process detailed below.
Firstly, the biological process (BP) ontology dataset within GO was examined using Quick GO (refer to Box 1) in order to identify all BP terms associated with the genes under investigation, hereafter called originally selected GO terms (OGO terms). No restrictions were given on the type of evidence codes used for the annotation of the OGO terms. Secondly, a web based tool called Generic Gene Ontology (GO) Term Mapper (http://go.princeton.edu/cgi-bin/GOTermMapper) was used to identify common biological processes among our gene list. Protein IDs of genes (**Table 2**) were used as input data and the biological process ontology was selected. The set of GO Slim terms used to obtain common BP terms was the Generic GO Slim for human GO annotations. GO Slim terms are GO terms that are very broad and therefore parent terms of OGO terms. Thirdly, common biological processes were identified based on having three or more genes associated within a domain from the 21 genes found in **Table 2**, resulting in a frequency of occurrence of about 14% or more. Identifying domains with a minimum of three genes aids in forming an in-depth GO network. This is because with three genes there is sufficient amount of associated GO terms to represent a meaningful network. Only OGO terms associated with these common processes are carried forward to the next step. Lastly, biological domains selected should be a child term which is three or more child term away from its parent GO term “Biological Process.” GO terms higher up (closer to the GO term “Biological Process”) in the hierarchy of the ontology, are very broad encompassing many genes resulting a biological domain that are not well-defined, therefore potentially introducing noise.
Based on the outcome of the three steps outlined above, we grouped all child terms that derived from parent terms in the domains of vesicle-mediated transport, organic substance transport and ion transport. These three parent terms were found to be under the common network of “transport system,” which was identified as a common biological process. GO terms that fell under the network “transport system” were the fifth level child terms from the Biological Process parent GO term. Refer to Box 1 under Gene Ontology section for child and parent terminology. In order to benchmark our approach in the selection of common biological processes, INRICH (Lee et al., 2012) was used as an alternative, objective, method to derive the common biological process domains. However, no significant results were identified to take forward to sFDR. The INRICH process is defined in the Supplementary Section 1.
**Step 3:** Cytoscape 3.2.0 (refer to Box 1) was used to visualize the biological process network “transport system,” and parent GO terms from the OGO terms were extracted to contextualize this network. As expected, the networks were overly complex and contained much extraneous information. To remedy this, an algorithm was developed to effectively reduce redundant data in order to create an effectively “pruned” network. This is accomplished by using building and pruning techniques based on the relationships of OGO terms. Figures 2–**4** demonstrate different stages of this algorithm with the OGO terms in green boxes. Figure 2 shows a subsection of GO terms in the complete “transport system” network before pruning of the data. Figures 3A–**D** display how specific criteria were used to remove non-targeted GO terms. Figure 4 shows the final pruned data of the transport system.

Box 1 **Gene ontology**

Gene Ontology (http://geneontology.org/) is a publically available, free, ontology database that describes protein function (Ashburner et al., 2000). Gene products—proteins—are classified and grouped in three main ontologies: cellular components (CC) where the protein is located within subcellular compartments, molecular functions (MF) indicates the specific function of the gene is carried out in normal conditions and biological processes (BP) which describes the processes a protein is involved in (e.g.,: neurogenesis). The ontology follows a hierarchical order and there are defined relationships between the GO terms. In the ontology structure, terms at the top represent general or broad concepts, whereas terms near the bottom represent more detailed processes. Therefore, if a term has terms subordinate to it, it is referred to as a “parent” term. Similarly, if a term has other terms superior to it, then it is referred to as a “child” term. Both manual and automatic annotations of proteins are available in the GO database. Automatic annotations are inferred from electronic annotations and are not manually reviewed by a curator. In manual annotations, a curator reviews primary articles to generate annotations, and each annotation is based on experimental data referenced to a PubMed ID. The documentation for manual curation can be found at http://geneontology.org/page/annotation, and an example of annotations created by the authors can be found in the Alzheimer's University of Toronto dataset at http://www.ebi.ac.uk/QuickGO/GAnnotation?source=Alzheimers_University_of_Toronto Quick GO (http://www.ebi.ac.uk/QuickGO/) is a web based tool used to extract data from the GO database.

**Cytoscape**

Cytoscape is an open source software platform visualization tool used to integrate data into complex networks of molecular interaction and biological pathways [(Saito et al., 2012), http://www.cytoscape.org/]. See Figure 4 as an example of a biological network.

The following criteria were used to select the child and parent GO terms.

- When extracting the ontology of the OGO terms using Cytoscape, child terms are automatically selected. Therefore, to simplify the ontology networks, child terms were removed. Orange terms in Figure 3A represent extra child terms of the OGO terms, which are not needed in the network. For example the GO term “clathrin-mediated endocytosis” has two child terms “clathrin-mediated extracellular exosome endocytosis” and “clathrin-mediated endocytosis of virus by host cell.” These terms are not necessary because the genes from step 1 have not been associated with these GO terms. (Figure 3A).
- If more than one parent term is identified for an OGO term, then a common parent term, which is shared by most of the OGO terms, is chosen. As an example, the term “receptor internalization” has two parent terms, namely, “receptor metabolic process,” and “receptor-mediated endocytosis.” In Figure 3B the term “receptor metabolic process,” displayed in a pink box, is removed because the alternate parent term, “receptor-mediated endocytosis,” is a parent term to both the selected GO terms “clathrin-mediated endocytosis” and “receptor internalization.” (In this case receptor-mediated endocytosis is an OGO term but the same criteria is followed if receptor-mediated endocytosis was not an OGO term).
- A positive or negative regulation child term will have two types of parents. As an example, we will investigate the term “negative regulation of receptor-mediated endocytosis.” The first parent will be the term it regulates (“receptor-mediated endocytosis”) and the second parent would likely be a term that has “regulation” as a key word in the term name, for example, “regulation of receptor-mediated endocytosis” could be a candidate. Therefore the parent term that is regulated was selected, in this case the term “receptor-mediated endocytosis,” and the parent term that regulates a biological process but does not specify positive or negative regulation (“regulation of receptor-mediated endocytosis”) is removed—shown in a yellow box – because the child term is more specific in terms of explaining how it is regulating the parent term (e.g., negative regulation of receptor-mediated endocytosis), Figure 3C.
  **Step 4:** Quick GO was used to extract all the genes that are associated to the OGO terms (as displayed in Figure 4 in green boxes) in the pruned “transport system” network. SNPs from these genes were extracted from the ENIGMA2 and ADNI1 (Supplementary Section 2) dataset using a reference file containing the start and end positions of the transcribed gene portion according to the Homo sapiens build 37 protein and coding genes from National Center for Biotechnology Information (NCBI). This list of SNPs formed the priority list for sFDR.

### ENIGMA2 dataset

The ENIGMA consortium is composed of a network of international researchers collaborating together on large scale genetic and MRI analysis (Thompson et al., 2014). Datasets from 70 institutions worldwide are utilized in meta-analysis studies for imaging genetics. The initial sample size for ENIGMA1 included a total of 24,997 individuals. The first large scale project for ENIGMA was investigating common genetic variants from case controls samples for neuropsychiatric disorders (depression, anxiety, Alzheimer's disease and schizophrenia) in association with hippocampal and intracranial volumes.

In this paper we use summary statistics of the ENIGMA2 dataset which includes ENIGMA1 individuals and new individuals increasing the sample size to 30,717 individuals from 50 cohorts. The summary statistic contained a total of 6,570,616 SNPs. In this large scale meta-analysis, association testing was done with imputed GWAS data and volumes of major subcortical structures including: intracranial volume, nucleus accumbens, amygdala, caudate, pallidum, putamen, thalamus, and hippocampus (Hibar et al., 2015). In this paper we used summary statistic of GWAS association with hippocampal volumes. Protocols for genetic and MRI quality control and analysis for ENIGMA2 are available online at http://enigma.ini.usc.edu/protocols/.

### sFDR

Fixed FDR strategies are used to control FDR in a group of tests. In sFDR, SNP *p*-values from the association analysis are grouped into distinct strata, one or more of which are believed to have a higher prior probability of being associated with the trait of interest (Sun et al., 2006). The association *p*-values of each SNP are transformed to *q*-values and FDR is controlled separately within each strata. To control the FDR at a given level—for example 10%—the null hypothesis is rejected when tests have a *q*-value equal to or less than the specified threshold (0.1). This method increases the power to identify true associations if one of the strata is enriched with associated variants. When the strata aren't enriched, the method is still robust. Two SNP strata were formed in our data. All SNPs in the genes associated to the OGO terms (Figure 4) from the pruned “transport system” network formed one, high priority, strata (274,272 SNPs), and all the remaining SNPs formed the other (6,296,344 SNPs) in our non-priority stratum. Association *p*-values from ENIGMA2 summary statistics were merged with each corresponding SNPs in each strata (priority and non-priority list) for sFDR. A Perl script was used to analyze priority and non-priority SNPs (http://www.utstat.toronto.edu/sun/Software/SFDR/).

### Null models: prostate cancer and random seed gene list

To address the concern of overfitting to a generic biological signal in GO, we tested our approach against a null model. We chose prostate cancer since cancer and AD generally have different biological characteristics (Behrens et al., 2009). A seed list of 21 genes associated with prostate cancer was identified from Stadler et al. (2010) review (Stadler et al., 2010). In addition, three random gene lists, were generated using a random number generator of Entrez IDs in R to randomly select for 24 genes (similar to the number of genes in our seed gene list for AD). For each gene list four biological domains common to the genes within the list were selected. We then undertook sFDR control with these gene strata in the ENIGMA2 GWAS hippocampal volume dataset.

### Datasets

The two data sets used in this study are the ADNI1 data and ENIGMA2 summary statistics.

The ENIGMA consortium is composed of a network of international researchers collaborating together on large scale genetic and MRI analysis. ENIGMA2 dataset was used in this study, which includes also ENIGMA1 individuals with a total sample of 30,717. In this large scale meta-analysis, association testing was done with imputed GWAS data and volumes of major subcortical structures such as hippocampal volume (Hibar et al., 2015). To obtain access to the ENIGMA2 summary statistics, a “Data Agreement for ENIGMA2 Download form” was completed online at http://enigma.ini.usc.edu/publications/enigma-2/data-agreement-for-enigma2-download/. More information of the ENIGMA dataset can be found in Section ENIGMA2 Dataset.

The ADNI database has been established in 2003 to facilitate the development of methods for biomarker investigation in order to enable detection of Alzheimer's disease at earlier stages. The ADNI database contains different information including neuroimaging, clinical, and genome-wide SNPs data. According to the ADNI protocol, subjects are diagnosed as cognitively normal (CN), mild cognitive impairment (MCI), or Alzheimer's disease (AD), based on the severity of their condition, and are recruited from Canada and the United States. To obtain access to the ADNI data “ADNI Data Use Agreement,” had to be completed online at http://adni.loni.usc.edu/data-samples/access-data/. More information of the ADNI dataset can be found in Supplementary Section 2.1.

## Results

In this paper we will focus on the results obtained by prioritizing genes from the transport system GO network using the AD seeded gene list with the ENIGMA2 dataset (known as our AD model) which improved sFDR values compared to results obtained with the ADNI1 dataset. More information of association analysis and sFDR analysis using ADNI1 data can be found in the Supplementary Section 2.2.

### AD model: SNP selection

The following results for selection of priority genes are shown corresponding to each step presented in the methods section. In total 24 genes were used as our original gene list in which GO terms associated to these gene were extracted to form the “transport system” network. The extraction of additional genes associated with OGO terms increased our gene list from 24 to 1727 priority genes. Below, we discuss in detail the results from each step when selecting for priority list of genes.

**Step 1:** From the 21 loci identified in Lambert et al., (2013) in association with AD, 10 were already known through previous GWAS and 11 novel loci were found (Table 1). *APP, PSEN1*, and *PSEN2* were also added to the gene list.
**Step 2:** Common biological processes within the gene list were identified using GO and Generic Gene Ontology (GO) Term Mapper web based tool. INRICH was used as an alternative objective method, but significant results were not found. Regardless, results from INRICH are found in the Supplementary Section 1.2. The GO database was accessed on July 2nd, 2015. In the GO database all genes from the list had BP GO terms annotated to them except the gene Membrane-spanning 4-domains subfamily A member 6A (*MS4A6A*). Table 2 shows the common BP domains associated with the 21 genes. In this study we focused on the “transport system” network (Figure 4), which included many genes from our original list. The network can be broken down into sub-domains with key GO terms in the areas of vesicle-mediated transport, organic substance transport, and ion transport. For example, in the domain “vesicle-mediated transport”, Phosphatidylinositol-binding clathrin assembly protein (*PICALM*) has been associated with GO terms “receptor-mediated endocytosis” and “clathrin-mediated endocytosis.”
**Step 3:** Cytoscape visualization of the transport network is shown in Figure 4.
**Step 4:** The list of genes associated with the OGO terms from the pruned “transport system” network included 1727 genes, after removal of all non-autosomal genes 1671 genes remained and formed our stratum for sFDR. Supplementary Excel Table S1 shows a list of all priority genes with chromosome number, start and end position and gene symbol. Furthermore Supplementary Excel Table S2 contains all 274, 272 SNPs from 1671 genes used for sFDR.

**Table 1:** **Top genes associated with AD from the Lambert et al. (2013) meta-analysis**.

**(A) 10 loci known to be associated with Alzheimer's disease**	
**Gene Symbol**	**Gene Name**
APOE	Apolipoprotein E
BIN1	Myc box-dependent-interacting protein 1
CLU	Clusterin (Apolipoprotein J)
ABCA7	ATP-binding cassette sub-family A member 7
CR1	Complement receptor type 1
PICALM	Phosphatidylinositol-binding clathrin assembly protein
MS4A6A	Membrane-spanning 4-domains subfamily A member 6A
CD33	Myeloid cell surface antigen CD33
CD2AP	CD2-associated protein
EPHA1	Ephrin type-A receptor 1
**(B) 11 new loci associated Alzheimer's disease**	
HLA	Human leukocyte antigen class II histocompatibility antigen
SORL1	Sortilin-related receptor
PTK2B	Protein-tyrosine kinase 2-beta
SLC24A4	Sodium/potassium/calcium exchanger 4
NYAP1	Neuronal tyrosine-phosphorylated phosphoinositide-3-kinase adapter 1
CELF1	Encode CUGBP, Elav-like family member 1 region
NME8	Thioredoxin domain-containing protein 3
FERMT2	Fermitin family homolog 2
INPP5D	Phosphatidylinositol 3,4,5-trisphosphate 5-phosphatase 1
MEF2C	Myocyte-specific enhancer factor 2C
CASS4	Cas scaffolding protein family member 4

**Table 2:** **Common GO Biological Process domains of gene hits from the Lambert et al. (2013) meta-analysis**.

**Gene**	**Protein ID**	**Transport System**	**Steroid and cholesterol metabolic process**	**Immune system process**	**Cell membrane processes and Cell migration**	**Nervous system development and Synaptic transmission**	**Regulation of calcium-mediated signaling**
DRB5	Q30154			X			
SORL1	Q92673	X	X		X		
PTK2B	Q14289			X	X	X	X
SLC24A4	Q8NFF2	X					X
NYAP1	Q6ZVC0					X	
MADD	Q8WXG6				X		
NME8	Q8N427				X		
FERMT2	Q96AC1				X		
INPP5D	Q92835			X			
MEF2C	Q06413					X	
CASS4	Q9NQ75						
APOE	P02649	X	X		X	X	X
BIN1	O00499	X			X		
CLU	P10909		X	X			
ABCA7	Q8IZY2	X	X	X			
CR1	P17927			X			
PICALM	Q13492	X		X		X	
MS4A6A	Q9H2W1						
CD33	P20138			X	X		
CD2AP	Q9Y5K6				X		
EPHA1	P21709			X	X		

### Summary statistics of ENIGMA2 GWAS meta-analysis of hippocampal volume

*P*-values were extracted from summary statistics of association testing between SNPs and hippocampal volume from the ENIGMA2 dataset. Significant SNPs were identified as *p* < 5 × 10^−8^. For example the top significant SNP rs77956314 (*P* = 9.33 × 10^−11^) is located in the intergenic region near the gene, activator of apoptosis harakiri (HRK) on chromosome 12. (For more information on association analysis results and Manhattan plot please refer to Hibar et al., 2015).

### AD model: sFDR results using ENIGMA2 GWAS meta-analysis of hippocampal volume

In total there were 274, 272 SNPs in our priority stratum (transport system) and 6,296,344 SNPs in our non-priority stratum of our AD model. SNPs in the transport system priority group showed a sFDR between 10 and 20% in gene regions Sodium-driven chloride bicarbonate exchanger (SLC4A10), Potassium voltage-gated channel subfamily H member 7 (KCNH7), Cationic amino acid transporter 2 (SLC7A2), Zinc transporter ZIP1 (SLC39A1), and Protein PTHB1 (BBS9). FDR was used as a benchmark for sFDR by comparing the *q*-values. The top SNPs from our priority group rs117831534 in gene region Calcineurin B homologous protein 3 (TESC) performed the same in FDR and sFDR however SNP rs118025365 performed slightly better in FDR than sFDR (Table 3). The remaining SNPs in the transport system priority list showed improved *q* sFDR values than FDR alone. For example a set of SNPs in the SLC4A10 gene has a *q* sFDR value of 0.118 (rs12472555, rs4500960, rs6707646, rs7580486, and rs7604885) whereas FDR *q*-value was 0.152. Furthermore SNPs rs10048805 and rs10172470 showed greater improvement of sFDR with a *q*-value of 0.165 compared to FDR of 0.304.

**Table 3:** **Top 10 sFDR results from summary statistics on ENIGMA2 GWAS meta-analysis of hippocampal volume**.

**Chromosome Number**	**SNP**	**Base position**	***p*-value**	***q*_valueFDR**	**Rank FDR**	***q*_value sFDR**	**Rank sFDR**	**Gene**
12	rs117831534	117506632	4.91E-07	0.068	47	0.067	47	TESC
12	rs118025365	117477082	4.00E-07	0.058	45	0.067	46	TESC
2	rs12472555	162816728	3.04E-06	0.152	103	0.118	53	SLC4A10
2	rs4500960	162818621	2.77E-06	0.152	91	0.118	52	SLC4A10
2	rs6707646	162808640	2.02E-06	0.152	70	0.118	49	SLC4A10
2	rs7580486	162810159	2.46E-06	0.152	83	0.118	51	SLC4A10
2	rs7604885	162806408	2.30E-06	0.152	80	0.118	50	SLC4A10
2	rs4664442	162828001	3.58E-06	0.152	150	0.122	55	SLC4A10
2	rs10048805	163466462	2.20E-05	0.304	473	0.165	216	KCNH7
2	rs10172470	163476863	1.66E-05	0.304	359	0.165	202	KCNH7

*P-value is the associated significance between the SNP and phenotype (hippocampal volume). Significant SNPs at a GWAS level is p < 5 × 10^−8^. The sFDR q-value controls the false discovery rate; the q-value is the adjusted p-value. “Rank” is the order of SNPs based on sFDR q-values from a total of 6,570,616 SNPs*.

### AD model comparison against null model SFDR results

To ensure our approach of using GO to prioritize genes in our AD model was not simply resulting in false positives we used the ENIGMA2 GWAS data and selected set of genes not related to AD. As our null models we chose prostate cancer and three random gene lists which were classified into biological domains using GO. Comparing the AD model against the prostate cancer model and three random null models, SNPs within each biological domain within the null models did not show sFDR values below 20% for each of the four biological domains selected within each gene list (Supplementary Excel Tables S3–S6). Whereas SNPs within the AD model showed sFDR value less than 20% (as described in Section AD Model: sFDR Results Using ENIGMA2 GWAS Meta-Analysis of Hippocampal Volume). In the gene seed list of the null models, biological domains selected were: anatomical structure formation involved in morphogenesis, cell differentiation, cellular component assembly and signal transduction. Furthermore, there was no reduction in sFDR *q*-values when compared to FDR within each biological domain from both the prostate cancer and random gene seed lists.

## Discussion

In contrast to existing approaches, our novel method provides a systematic integration framework for previous knowledge with the GO database. Alternatives such as Aligator and INRICH both rely on the identification of over-represented GO categories among significant hits. These approaches use GO as the last step to identify which biological domains are enriched based on significant genetic variants providing results relating to the whole domain and not individual variants. In contrast, we identify and adapt relevant categories based on GO and use sFDR to increase power while controlling for multiplicity, the method provides *q*-values for individual variants. A direct comparison between our approach to tools such as INRICH and Aligator is therefore difficult. However we have compared the intermediate step of manually selecting biological domains use in our approach to a gene enrichment tool, INRICH (detail found in Supplementary Section 1.1).

Results from sFDR are promising, in the ENIGMA2 dataset *q*-values of sFDR ranged in the 10–20%. Furthermore SNPs in the transport priority list using the ENIGMA2 data showed improved q sFDR values than FDR (Table 3) indicating a potential link between the genes in our pruned transport system network in connection to hippocampal volume. SNPs with 10–20% q sFDR from the ENIGMA2 data are found in gene regions SLC4A10, KCNH7, SLC7A2, SLC39A1, and BBS9. However, the SNPs are within introns region of (non-cording regions) the gene therefore the role a SNP will have on a gene function is unclear. RegulomeDB was used to investigate the role of these SNPs in non-coding regions (Boyle et al., 2012), however no further evidence of functionality was provided. The SNPs we identified may just be tagging SNPs hence the causal variants may be within genes that are close by. For example, the SLC4A10 gene is a sodium/bicarbonate co-transporter for intracellular chloride exchange. It plays a key role in regulating intracellular and extracellular pH for synaptic transmission, nerve stimulation, and enzyme activities and it is expressed in the brain (Fang et al., 2010). During aging and or ischemia, accumulation of acidic metabolites decreases the pH which can affect the activity of enzymes related to APP processing affecting the amyloid plaque formation in AD (Song et al., 2003; Xue et al., 2006). Another genes of interest to AD is KCNH7 which is a zinc transporter also known as ZIP1. Tau protein phosphorylation and aggregation is affected by changes in Zn^+2^ ion levels which in turn affect neurofibrillary tangles (Mo et al., 2009) and amyloid plaque formation (Lee et al., 2002).

In our AD model, stratified SNPs extracted from GO within the transport system showed sFDR *q*-values less than 20%, using the ENGIMA dataset which was not seen within each biological domain in the null models (prostate cancer and random seed gene lists). Comparison of AD model against the results from the null models addresses the concern of overfitting in our approach because if our approach was identifying random errors or noise as significant sFDR *q*-values, the null model will also identify random SNPs as being significant. Furthermore the purpose of applying sFDR is to investigate if a subset of SNPs based on common biological characteristics has more significant *q*-values compared to a standard FDR. In our AD model the sFDR results for the selected biological process were more significant than that of the standard FDR results but the results were the same in the null datasets. Hence, stratification of SNPs alone did not make the *q*-values more significant. As we are unable to provide nested models of FDR and sFDR and we do not know which genes are actually truly involved in AD, we are unable to determine if sFDR is significantly better than FDR. Thus at this time our results are suggestive.

Some aspects of the process by which priority SNPs are selected for sFDR could be considered subjective and represent an area of active development for our algorithm. For example, often the associations in GWAS studies are designated to the most promising gene in the region from a biological standpoint, introducing bias in step 1. One approach to combat this phenomenon would be for the input list to include all genes within a high recombination region alongside the most significant hit. Selection of the common biological domains in step 2 can be performed in a variety of ways due to different tools available. In our approach we used a web based tool Generic Gene Ontology (GO) Term Mapper which decreases the subjectivity of identifying common biological domains. However because there are different GO Slim terms that can be used on a gene set, identified common biological domains in GO can differ. Other potential standard pathway approaches are INRICH or Aligator. We have performed pilot work using INRICH as outlined in the Supplementary text. In this example no pathways were identified, preventing us from pursuing this avenue. This is likely due to a lack of power to identify relevant pathways.

Another area of active development relating to SNP selection revolves around growing and pruning the network of terms utilized. As such, further pruning the GO network by focusing on BP GO terms annotated to genes specific to one brain region, such as the hippocampus, or neuronal cell type within such structures may be crucial. Biological processes associated with structural information have recently begun to be captured in GO (Huntley et al., 2014). Therefore, when genes are annotated to BP GO terms, additional information on where the biological process is occurring can be recorded. As a result, filtering the data and looking at BP GO terms occurring in neuro-anatomical cells in region of the hippocampus may help in further pruning the network.

Both automatic and manual curation was used to assign GO terms to the genes in question. Automatic curation is the result of machine learning algorithms, and the terms assigned tend to be much broader than the manually curated ones and adds a potential source of noise to our priority SNPs stratum. In the example analysis presented here the inclusion of these sub-optimal classifications was necessary due to the limited manual annotation of the loci observed in Lambert et al. (2013) yet we acknowledge the shortcomings of this approach and advise the prioritization of manually curated GO data. To further address the issue we are in the process of manually curating the list of 21 loci associated with AD.

In conclusion, this article introduces the use of GO, an online database, as a novel method to efficiently prioritize data for sFDR multiple testing control. We take advantage of how the GO terms in the biological process ontology relates to each other in a hierarchical order ontology and we capture the different properties of a particular biological process that is impaired in a disease.

In particular we applied this method to a GWAS of hippocampal volume in the ENIGMA2 and ADNI1 dataset. Our method has the potential to improve the identification of genes in imaging-genetic studies; further development along the lines described above could increase this ability.

## Author contributions

SP performed quality control and imputation on GWAS data, developed the Gene Ontology network and performed the association analysis between GWAS data and mean hippocampal volume. MP carried out automatic and manual hippocampal segmentation and quality control on the segmentation. SP, JK, MC conceptualized the design of the study. All authors wrote and edited the manuscript.

### Conflict of interest statement

The authors declare that the research was conducted in the absence of any commercial or financial relationships that could be construed as a potential conflict of interest.
