# Supplementary material for: Gene Prioritization for Imaging Genetics Studies Using Gene Ontology and a Stratified False Discovery Rate Approach
Source: Front Neuroinform. 2016 Apr 7;10:14. doi: 10.3389/fninf.2016.00014 (PMC4823264; doi:10.3389/fninf.2016.00014)
Supplement: Supplementary file 7 [file DataSheet1.PDF]

Supplementary Material

Gene prioritization for imaging genetics studies using Gene Ontology

Sejal Patel[1,2*], Min Tae M. Park[3,4], The Alzheimer's Disease Neuroimaging Initiative[a], M. Mallar Chakravarty[3,5#], Jo Knight[1,2,6,7#]

[1] Campbell Family Mental Health Research Institute, Centre for Addiction and Mental Health, Toronto, ON, Canada
[2] Institute of Medical Science, University of Toronto, Toronto, ON, Canada
[3] Cerebral Imaging Centre, Douglas Mental Health University Institute, McGill University, Verdun, QC, Canada
[4] Schulich School of Medicine and Dentistry, Western University, London, ON, Canada
[5] Department of Psychiatry, McGill University, Montreal, QC, Canada
[6] Department of Psychiatry, University of Toronto, Toronto, ON, Canada
[7] Dalla Lana School of Public Health, Biostatistics Division, University of Toronto, Toronto, ON, Canada

*** Correspondence:**

Sejal Patel
Campbell Family Mental Health Research Institute
Centre for Addiction and Mental Health
250 College Street
Toronto, ON, M5T 1R8, Canada
Sejal.Patel@camh.ca

[#]M. Mallar Chakravarty and Jo Knight are co-senior authors.

[a] Data used in preparation of this article were obtained from the Alzheimer's Disease Neuroimaging Initiative (ADNI) database (adni.loni.usc.edu). As such, the investigators within the ADNI contributed to the design and implementation of ADNI and/or provided data but did not participate in analysis or writing of this report. A complete listing of ADNI investigators can be found at: http://adni.loni.usc.edu/wp-content/uploads/how_to_apply/ADNI_Acknowledgement_List.pdf.

1. INRICH

1.1. Methods: Alternative Step 2

Step 2 in our method approach is subjective because the investigator determines common processes by reviewing the data and hence it could be subject to bias. Furthermore, it relies on genes cited in previous GWAS which may not be causal and may simply be the best candidate gene within the associated region. To provide an overall systematic approach we implemented an alternate second step using an existing pathway tool INRICH (Lee *et al.*, 2012). INRICH determines if linkage disequilibrium (LD) independent associated regions show an enrichment of specified characteristics, primarily pathways defined by GO terms. We applied this tool to the summary statistics of the

primary GWAS analysis from the Lambert et al paper, available for download (http://www.pasteur-lille.fr/en/recherche/u744/igap/igap_download.php). Analysis requires a list of gene names from the entire genome and corresponding GO terms. We used a preformatted list of Entrez gene IDs and GO terms from the INRICH website; we manipulated the list of GO terms to only contain Biological Process terms with associated Entrez IDs. Parameters used to create independent interval file were the following: LD clumping with a measure of $r^2 \leq 0.5$ and p-value thresholds for SNPs in associated regions were set at $1x10^{-6}$ and $5x10^{-4}$. This is an alternate method for defining common biological process networks for selection of SNPs using steps 3-4.

1.2. Results: GO domains selection using INRICH

INRICH was used to investigate if the manual selection process of GO domains using gene hits from the Lambert et al could be automated. The results in INRICH did not show significant GO terms that fall under the domains selected manually. However the term, 'regulation of endocytosis' was selected with a corrected p-value of 0.37. This terms falls under the 'vesicle-mediated transport' and 'endocytosis' domain which was manually identified. Other GO terms that was identified but not significant was the 'complement activation classical pathway' with corrected p-value of 0.18.

2. Applying GO approach to ADNI 1 dataset

2.1. Method: ADNI1 Data

GWAS data and magnetic resonance imaging (MRI) neuroimaging data was obtained from the Alzheimer's Disease Neuroimaging Initiative (ADNI). Established in 2003 to facilitate the development of methods for biomarker investigation in order to enable detection of Alzheimer's disease (AD) at earlier stages, ADNI is a partnership between the National Institute on Aging, the National Institute of Biomedical Imaging and Bioengineering, the Food and Drug Administration, private pharmaceutical companies, and nonprofit organizations (http://adni.loni.usc.edu/ ; Michael W. Weiner, Principal Investigator). The ADNI database contains different information including neuroimaging, clinical, and genome-wide SNPs data. According to the ADNI protocol, subjects are diagnosed as cognitively normal (CN), mild cognitive impairment (MCI), or Alzheimer's disease (AD), based on the severity of their condition, and are recruited from Canada and the United States. We used the ADNI1 dataset "ADNI1: Complete 1Yr 1.5T" (Wyman *et al.*, 2013). 1.5T scanners (General Electric Healthcare, Philips Medical System or Siemens Medical Solutions) were used with the protocol described by (Jack *et al.*, 2008). Before quality control (QC), 817 Caucasian 1.5T MRI subject scans were obtained from the ADNI1 database. Of the 817 subjects, 757 had GWAS data and 662 passed quality control. **Figure S1** outlines the entire process followed, including SNP selection (**Figure S1A**) and the preparation and subsequent analysis of the genetic and imaging data (**Figure S1B**).

2.1.1. ADNI Imaging Data: Hippocampal Segmentation

Hippocampal segmentation was carried out in all 662 samples with GWAS data, using a modified multi-atlas algorithm known as the Multiple Automatically Generated Templates (MAGeT-Brain) algorithm (Chakravarty *et al.*, 2013; Pipitone *et al.*, 2014). The MAGeT Brain algorithm overcomes the limitations of model-based segmentation techniques, and avoids the requirement for larger atlas libraries typically required in more traditional multi-atlas segmentation strategies (Heckemann *et al.*, 2006; Collins and Pruessner, 2010) by bootstrapping the segmentation procedure using data from the participants being analyzed. The segmentation procedure consists of three steps. First, five high-resolution MRI atlases developed by our group were used as inputs (Winterburn *et al.*, 2013) and are used to automatically generate a "template library" based on a subset of the ADNI1 dataset using a model based segmentation procedure. For the purposes of this work we used a subset of subjects

consisting of 7 AD, 7 MCI and 7 CN subjects evenly distributed across an age range of 58-90 to model the anatomical variability across the ADNI1 dataset. Model-based segmentation is used to segment each of the subjects in the template library leading to a total of 5 candidate segmentations per subject. The next step proceeds much like a regular multi-atlas segmentation strategy, where each subject is nonlinearly matched to each of the subjects in the template library, yielding 105 (5 atlases × 21 templates) candidate segmentations for each subject. The last step is a voxel voting technique where a label at each voxel that is most frequently occurring is used for the final segmentation (Collins and Pruessner, 2010). All resultant segmentations were manually inspected by an expert rater and only those segmentations passing quality control were used in the analysis. Images not successfully segmented by the MAGeT Brain algorithm were segmented manually for use. All input atlases (http://cobralab.ca/atlases/Hippocampus.html) and source code for MAGeT-Brain are freely available online (https://github.com/CobraLab/MAGeTbrain). Nonlinear transformations were estimated using the ANTs algorithm (Avants *et al.*, 2008) and image processing steps were carried out using the MINC toolbox (http://www.bic.mni.mcgill.ca/ServicesSoftware/ServicesSoftwareMincToolKit).

2.1.2. ADNI1 Genetic Data: Quality Control

Quality control (QC) was performed on the ADNI 1 GWAS data (N=757) using PLINK (version 1.07, http://pngu.mgh.harvard.edu/~purcell/plink/ (Purcell *et al.*, 2007)). In addition R (http://www.r-project.org/) was used to visualize the results. Individuals with discordant sex information, high level of missing data (> 2%) and heterozygosity rates greater than 3 standard deviations from the mean were removed from the sample. One of each pair of individuals displaying a high level of pair-wise identity by descent (IBD > 0.185) were also removed. In addition, SNPs with minor allele frequency (MAF) <1% and Hardy-Weinberg equilibrium ($p < 1x10^{-7}$) were removed. After QC, 662 individuals remained in the analysis set. Multidimensional scaling (MDS) was performed in PLINK using HapMap3 (Altshuler *et al.*, 2010) as a reference panel. When the population is compared with the CEU (CEPH - Utah residents with ancestry from northern and western Europe), YRI (Yoruba in Ibadan, Nigeria), JPT (Japanese in Tokyo, Japan), TSI (Tuscans in Italy) and CHB (Han Chinese in Beijing, China) ancestry, the sample clustered around CEU and TSI sample. MDS was subsequently carried out with the ADNI1, CEU, TSI and Jewish ancestry samples and aligned completely with the later three samples (**Supplementary Figure S2**). The Jewish ancestry sample was made available by Mark Silverberg.

2.1.3. Data Preparation, Pre-Phasing and Imputation

The GWAS data was based on UCSC, (University of California, Santa Cruz) build 36 reference (Lander *et al.*, 2001), and the liftover tool available from the NCBI (http://genome.ucsc.edu/cgi-bin/hgLiftOver) was used to convert each SNP location to build 37. SHAPEIT 2.0 ((Delaneau *et al.*, 2012), https://mathgen.stats.ox.ac.uk/genetics_software/shapeit/shapeit.html) was used to pre-phase the haplotypes of the GWAS data after QC. Imputation was performed on the pre-phased data using Impute2 ((Marchini *et al.*, 2007), https://mathgen.stats.ox.ac.uk/impute/impute_v2.html) for the autosomal chromosomes with the 1000 Genome (March 2012) data as a reference. SNPs with info values of equal and greater than 0.5 and MAF > 0.05 were retained for analysis.

2.1.4. Association of Hippocampal Volume with GWAS Data

SNPTEST 2.5 ((Marchini *et al.*, 2007), https://mathgen.stats.ox.ac.uk/genetics_software/snptest/snptest.html) was used to examine associations between hippocampal volumes with both imputed and genotyped SNPs. Covariates used in the analysis were gender, age, first dimension from MDS to control for population structure, baseline diagnoses (CN, MCI, or AD), APOE status because *APOE* e4 carriers have a higher risk of

developing AD (Farrer *et al.*, 1997) and intracranial volume to correct for variation in brain sizes within individuals in the sample. Three phenotypes were investigated: left hippocampal volume, right hippocampal volume, and mean (of the left and right) hippocampal volume. Frequentist association testing was undertaken for each phenotype, with a 'method' option in place to control for genotype uncertainty in the association test.

2.1.5. Stratification of SNPs

Fixed FDR strategies are used to control FDR in a group of tests. In sFDR, SNP p-values from the association analysis are grouped into distinct strata, one or more of which are believed to have a higher prior probability of being associated with the trait of interest (Sun *et al.*, 2006; Sun *et al.*, 2012). The association p-values of each SNP are transformed to q-values and FDR is controlled separately within each strata. To control the FDR at a given level – 10% in this analysis – the null hypothesis is rejected when tests have a q-value equal to or less than the specified threshold (0.1). This method increases the power to identify true associations if one of the strata is enriched with associated variants. When the strata aren't enriched, the method is still robust. Two SNP strata were formed in our data. All SNPs in the genes associated to the OGO terms (**Figure 4**) from the pruned "transport system" network formed one, high priority, strata (252,494 SNPs), and all the remaining SNPs formed the other (5,454,064 SNPs) in our non-priority stratum. Association p-values from SNPTEST were merged with each corresponding SNPs in each strata (priority and non-priority list) for sFDR. A Perl script was used to analyze priority and non-priority SNPs (http://www.utstat.toronto.edu/sun/Software/SFDR/).

2.2. Results: ADNI 1 Data

2.2.1. Quality Control of ADNI 1Imaging and GWAS data

After quality control (QC) of automatic hippocampal segmentations, 9 segmentations out of 662 subjects failed which were corrected though manual segmentation. For the ADNI1 GWAS data, the sample initially consisted of 757 individuals, and after QC the sample was reduced to 662 subjects. The number of SNPs in the GWAS data after QC was 529,623 from 620,901 original variants, of which 517,064 SNPs were on autosomal chromosomes. After imputation of the GWAS, data the number of SNPs typed increased to 17,418,272. After QC of imputed SNPs, 5,706,558 SNPs were used for the association analysis with mean hippocampal volume.

2.2.2. Association Analysis with ADNI 1 Hippocampal Volume

P-values from association testing between the SNPs and mean hippocampal volume did not result in any GWAS significant findings after correction for multiple testing (**Figure S3**). Some, however, approached significance (**Table S1**, SNPs with uncorrected p-values). For example rs72909661 in gene region Stearoyl-CoA desaturase 5 (*SCD5)* neared GWAS significance with an uncorrected p= 8.97×10^{-7}. The top 10 SNPs found within gene regions were: Autism susceptibility gene 2 protein (*AUTS2*; rs2158616; p= 1.16×10^{-6}), Transmembrane protein - family with sequence similarity 155 member A (*FAM155A*; rs1033880; p=4.42×10^{-6}) and long non-coding RNAs (LOC440173; rs11791915; p=1.76×10^{-6}). Testing with left and right hippocampal volumes as response variables resulted in no GWAS significant findings, as displayed in the supplemental materials.

Table S1. Top 10 SNPs within gene regions from association analysis of mean hippocampal volume from ADNI dataset

MAF represents minor allele frequency and p-value is the associated significance between the SNP and phenotype (mean hippocampal volume). Significant SNPs at a GWAS level are at $p < 5x10^{-8}$.

Chromosome Number	SNP ID	Base position	MAF	p-value	NCBI Gene ID	Gene Symbol
9	rs75592689	89700141	0.07	6.92E-07	494127	LOC494127
4	rs72909661	83680826	0.05	8.97E-07	79966	SCD5
7	rs2158616	70075454	0.10	1.16E-06	26053	AUTS2
7	rs2158617	70075447	0.10	1.23E-06	26053	AUTS2
7	rs2158618	70075402	0.10	1.60E-06	26053	AUTS2
9	rs11791915	89643751	0.08	1.76E-06	440173	LOC440173
9	rs11789129	89638584	0.07	2.42E-06	440173	LOC440173
13	rs1033880	108222156	0.44	4.42E-06	728215	FAM155A
13	rs9520494	108222992	0.44	6.00E-06	728215	FAM155A
13	rs9520495	108224804	0.44	6.79E-06	728215	FAM155A

2.2.3. ADNI1 sFDR Results

In total there were 252,494 SNPs in our priority stratum and 5,454,064 SNPs in our non-priority stratum. No q-values from the priority list (transport system stratum) reached the 0.05 threshold. In particular, SNPs in our priority list within gene regions: Juxtaposed with another zinc finger protein 1 (*JAZF1*), Acetyl-CoA carboxylase 2 (*ACACB*), Phosphatidylinositol 4,5-bisphosphate 3-kinase catalytic subunit delta isoform (*PIK3CD*), Acid ceramidase (*ASAH1*) and Type II inositol 3,4-bisphosphate 4-phosphatase (*INPP4B*) were ranked in the top 10 but with an sFDR q-value of 0.75 (**Table S2**).

Table S2. Top 10 sFDR results for mean hippocampal volume from ADNI1 dataset

P-value is the associated significance between the SNP and phenotype (mean hippocampal volume). Significant SNPs at a GWAS level is $p < 5x10^{-8}$. The sFDR q-value controls the false discovery rate; the q-value is the adjusted p-value. 'Rank' is the order of SNPs based on sFDR q-values from a total of 5,706,558 SNPs.

Chromosome Number	SNP	Base Position	p value	q_value sFDR	Rank FDR	q_value sFDR	Rank sFDR	Gene
7	rs10486573	28080493	0.000647	0.9245	3938	0.7501	129	JAZF1
12	rs10849921	109622240	0.001018	0.9502	6082	0.7501	167	ACACB
12	rs10849926	109638760	0.000689	0.9245	4229	0.7501	132	ACACB
1	rs11121477	9745716	0.001005	0.9502	6016	0.7501	165	PIK3CD
8	rs111683029	17932715	0.000868	0.9502	5147	0.7501	146	ASAH1
8	rs112284453	17932724	0.000868	0.9502	5144	0.7501	148	ASAH1
8	rs112928371	17932778	0.000868	0.9502	5145	0.7501	145	ASAH1
4	rs11943397	143617304	0.000589	0.9245	3619	0.7501	123	INPP4B
1	rs12022504	9745337	0.001055	0.9502	6276	0.7501	170	PIK3CD
1	rs12028984	9744804	0.00092208	0.9502	5508	0.7501	150	PIK3CD

Supplementary Figures

Figure S1. Method overview of both the selection of priority SNPs and association testing analysis between ADNI1 GWAS and imaging data. **(A)** Steps taken to select for priority SNPs. Gene hits from a meta-analysis by Lambert *et al.*, (2013) were used as a starting point (Step 1) and GO was then used to identify common biological processes within the gene hits (Step 2). Cytoscape was used to build and visualize common biological process networks -- in this case the “transport system” network was selected (Step 3 and Step 4). All genes from the selected GO terms in the network were extracted to form the priority list of SNPs. sFDR was then implemented with the priority SNPs. (**B)** Shows quality controls steps taken GWAS data and hippocampal imaging data. Association analysis was performed between imputed quality control (QC) GWAS data with QCed hippocampal segmentation.

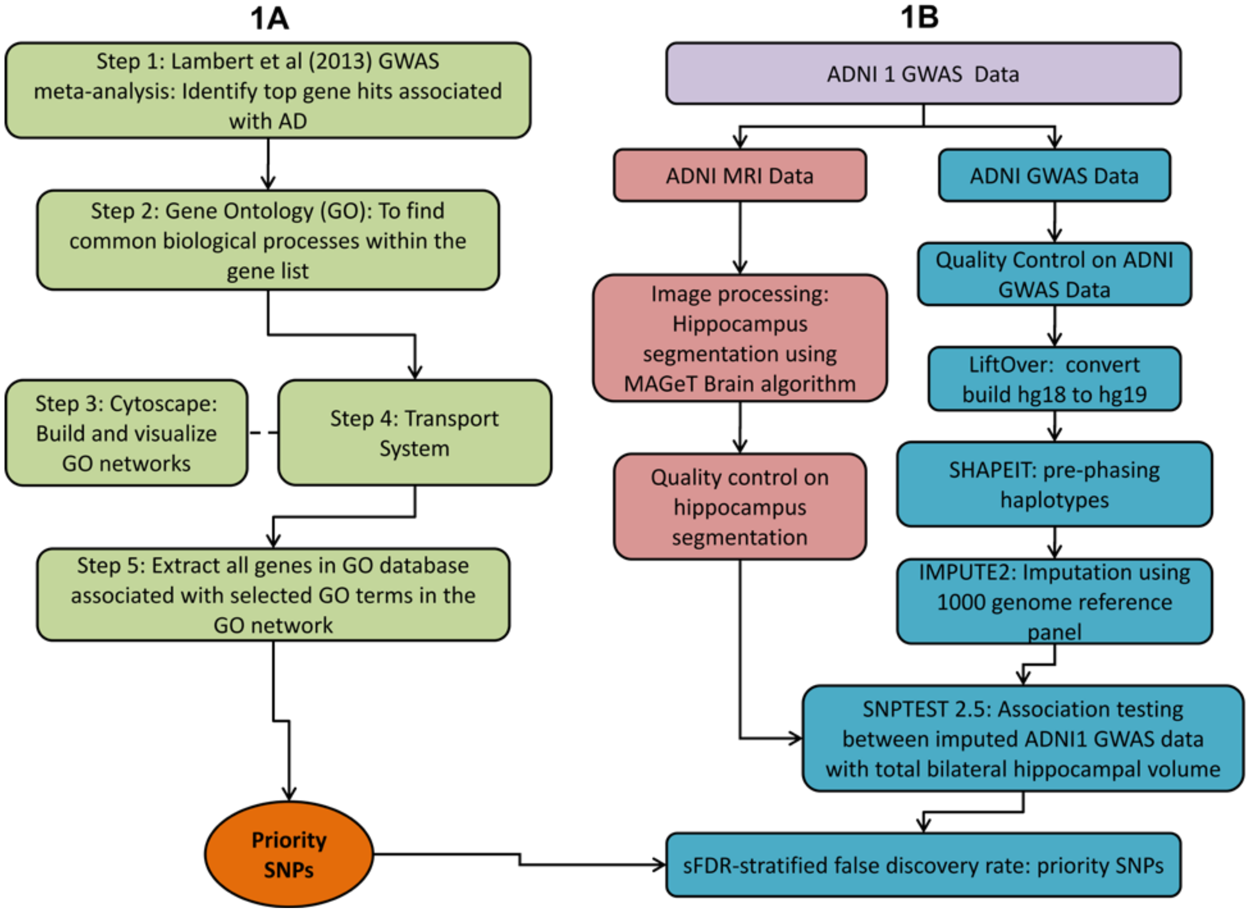

Figure S2. A multidimensional scaling plot of 662 subjects in the sample with HapMap3 ancestry, demonstrating cluster of different ancestry based on genetics data. Principle component 1 is plotted on x-axis and principle component 2 is plotted on the y-axis. ADNI1 sample (black) clustered with TSI (Tuscans in Italy, yellow), CEU (CEPH - Utah residents with ancestry from northern and western Europe, purple) and Jewish sample (red).

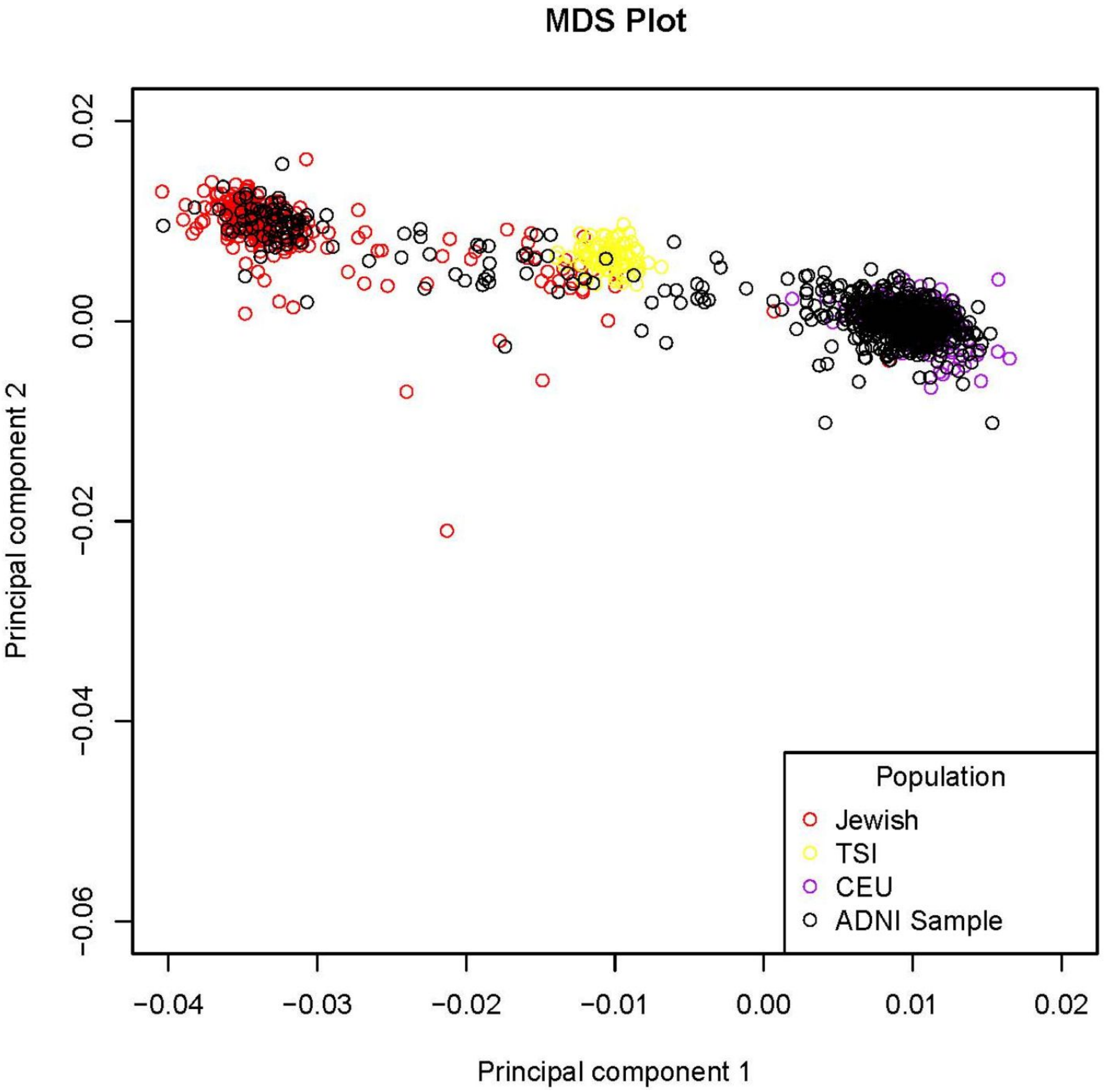

Figure S3. A Manhattan plot of imputed ADNI1 GWAS data. The x-axis represents the chromosomal location for each SNPs. The y-axis represents the log p-values of SNPs in association with AD. The red horizontal line represents the threshold for GWAS significant SNPs.

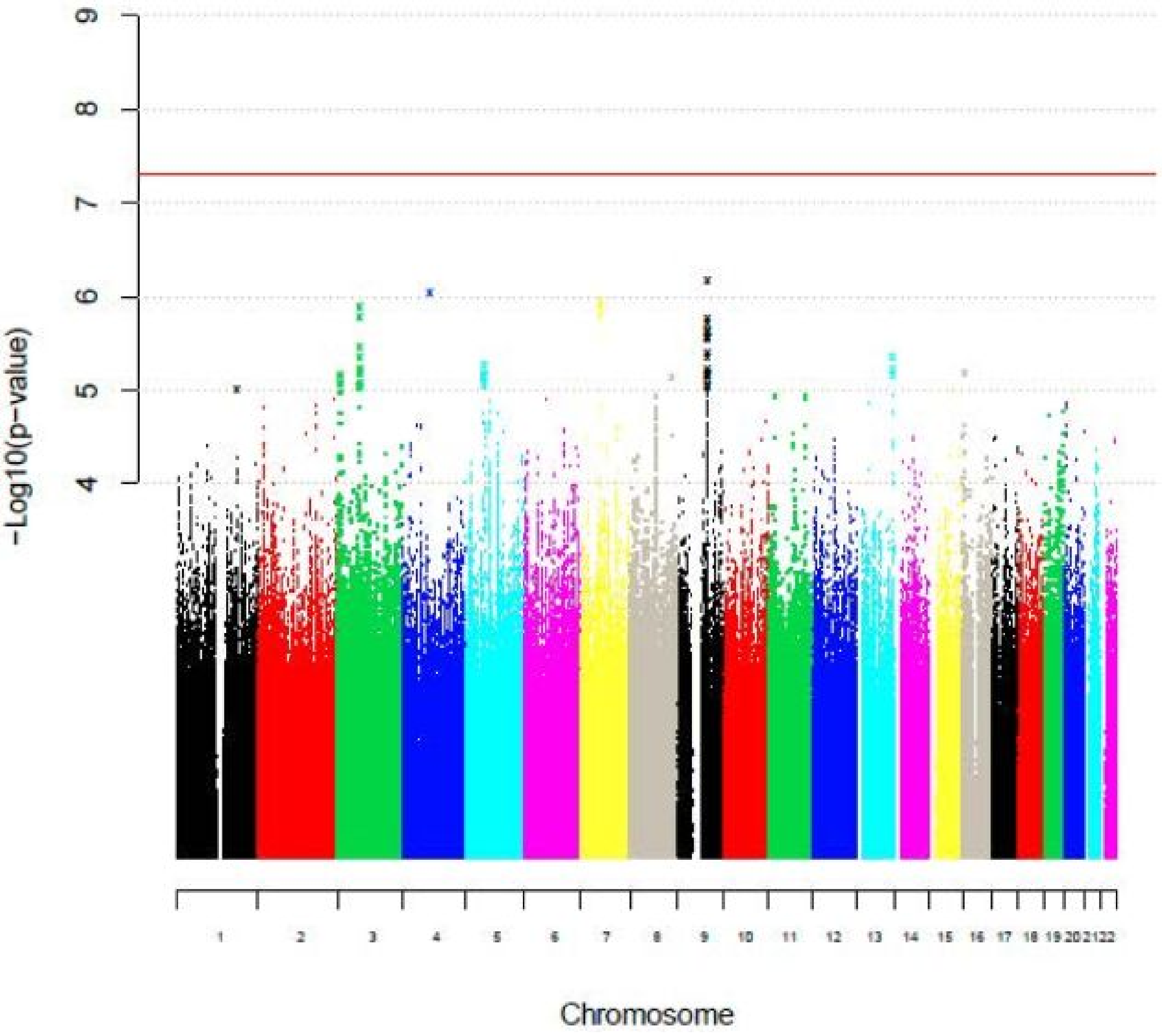

References

Altshuler, D.M., Gibbs, R.A., Peltonen, L., Dermitzakis, E., Schaffner, S.F., Yu, F., *et al.* (2010). Integrating common and rare genetic variation in diverse human populations. *Nature* 467, 52-58. doi: 10.1038/nature09298.

Avants, B.B., Epstein, C.L., Grossman, M., and Gee, J.C. (2008). Symmetric diffeomorphic image registration with cross-correlation: evaluating automated labeling of elderly and neurodegenerative brain. *Medical image analysis* 12, 26-41. doi: 10.1016/j.media.2007.06.004.

Chakravarty, M.M., Steadman, P., Van Eede, M.C., Calcott, R.D., Gu, V., Shaw, P., *et al.* (2013). Performing label-fusion-based segmentation using multiple automatically generated templates. *Human brain mapping* 34, 2635-2654. doi: 10.1002/hbm.22092.

Collins, D.L., and Pruessner, J.C. (2010). Towards accurate, automatic segmentation of the hippocampus and amygdala from MRI by augmenting ANIMAL with a template library and label fusion. *NeuroImage* 52, 1355-1366. doi: 10.1016/j.neuroimage.2010.04.193.

Delaneau, O., Marchini, J., and Zagury, J.F. (2012). A linear complexity phasing method for thousands of genomes. *Nature methods* 9, 179-181. doi: 10.1038/nmeth.1785.

Farrer, L.A., Cupples, L.A., Haines, J.L., Hyman, B., Kukull, W.A., Mayeux, R.*, et al.* (1997). Effects of age, sex, and ethnicity on the association between apolipoprotein E genotype and Alzheimer disease. A meta-analysis. APOE and Alzheimer Disease Meta Analysis Consortium. *JAMA* 278, 1349-1356.

Heckemann, R.A., Hajnal, J.V., Aljabar, P., Rueckert, D., and Hammers, A. (2006). Automatic anatomical brain MRI segmentation combining label propagation and decision fusion. *NeuroImage* 33, 115-126. doi: 10.1016/j.neuroimage.2006.05.061.

Jack, C.R., Jr., Bernstein, M.A., Fox, N.C., Thompson, P., Alexander, G., Harvey, D.*, et al.* (2008). The Alzheimer's Disease Neuroimaging Initiative (ADNI): MRI methods. *Journal of magnetic resonance imaging : JMRI* 27, 685-691. doi: 10.1002/jmri.21049.

Lander, E.S., Linton, L.M., Birren, B., Nusbaum, C., Zody, M.C., Baldwin, J.*, et al.* (2001). Initial sequencing and analysis of the human genome. *Nature* 409, 860-921. doi: 10.1038/35057062.

Lee, P.H., O'dushlaine, C., Thomas, B., and Purcell, S.M. (2012). INRICH: interval-based enrichment analysis for genome-wide association studies. *Bioinformatics* 28, 1797-1799. doi: 10.1093/bioinformatics/bts191.

Marchini, J., Howie, B., Myers, S., Mcvean, G., and Donnelly, P. (2007). A new multipoint method for genome-wide association studies by imputation of genotypes. *Nature genetics* 39, 906-913. doi: 10.1038/ng2088.

Pipitone, J., Park, M.T., Winterburn, J., Lett, T.A., Lerch, J.P., Pruessner, J.C.*, et al.* (2014). Multi-atlas segmentation of the whole hippocampus and subfields using multiple automatically generated templates. *NeuroImage* 101, 494-512. doi: 10.1016/j.neuroimage.2014.04.054.

Purcell, S., Neale, B., Todd-Brown, K., Thomas, L., Ferreira, M.A., Bender, D.*, et al.* (2007). PLINK: a tool set for whole-genome association and population-based linkage analyses. *American journal of human genetics* 81, 559-575. doi: 10.1086/519795.

Sun, L., Craiu, R.V., Paterson, A.D., and Bull, S.B. (2006). Stratified false discovery control for large-scale hypothesis testing with application to genome-wide association studies. *Genetic epidemiology* 30, 519-530. doi: 10.1002/gepi.20164.

Sun, L., Rommens, J.M., Corvol, H., Li, W., Li, X., Chiang, T.A.*, et al.* (2012). Multiple apical plasma membrane constituents are associated with susceptibility to meconium ileus in individuals with cystic fibrosis. *Nature genetics* 44, 562-569. doi: 10.1038/ng.2221.

Winterburn, J.L., Pruessner, J.C., Chavez, S., Schira, M.M., Lobaugh, N.J., Voineskos, A.N.*, et al.* (2013). A novel in vivo atlas of human hippocampal subfields using high-resolution 3 T magnetic resonance imaging. *NeuroImage* 74, 254-265. doi: 10.1016/j.neuroimage.2013.02.003.

Wyman, B.T., Harvey, D.J., Crawford, K., Bernstein, M.A., Carmichael, O., Cole, P.E.*, et al.* (2013). Standardization of analysis sets for reporting results from ADNI MRI data. *Alzheimer's & dementia : the journal of the Alzheimer's Association* 9, 332-337. doi: 10.1016/j.jalz.2012.06.004.
